# Dried blood spots as a source of anti-malarial antibodies for epidemiological studies

**DOI:** 10.1186/1475-2875-7-195

**Published:** 2008-09-30

**Authors:** Patrick H Corran, Jackie Cook, Caroline Lynch, Heleen Leendertse, Alphaxard Manjurano, Jamie Griffin, Jonathan Cox, Tarekegn Abeku, Teun Bousema, Azra C Ghani, Chris Drakeley, Eleanor Riley

**Affiliations:** 1 Department of Infectious and Tropical Diseases, London School of Hygiene & Tropical Medicine, London WC1E 7HT, UK; 2 Biotherapeutics Group, National Institute for Biological Standards & Control, South Mimms, Herts EN6 3QG, UK; 3 Joint Malaria Programme, KCMC Hospital, Box 2228, Moshi, Tanzania; 4 MRC Centre for Outbreak Analysis & Modelling, Department of Infectious Disease Epidemiology, Imperial College London, London W2 1PG, UK; 5 Department of Medical Microbiology, Radboud University Nijmegen Medical Centre, Nijmegen, the Netherlands

## Abstract

**Background:**

Blood spots collected onto filter paper are an established and convenient source of antibodies for serological diagnosis and epidemiological surveys. Although recommendations for the storage and analysis of small molecule analytes in blood spots exist, there are no published systematic studies of the stability of antibodies under different storage conditions.

**Methods:**

Blood spots, on filter paper or glass fibre mats and containing malaria-endemic plasma, were desiccated and stored at various temperatures for different times. Eluates of these spots were assayed for antibodies against two *Plasmodium falciparum *antigens, MSP-1_19 _and MSP2, and calculated titres used to fit an exponential (first order kinetic) decay model. The first order rate constants (*k*) for each spot storage temperature were used to fit an Arrhenius equation, in order to estimate the thermal and temporal stability of antibodies in dried blood spots. The utility of blood spots for serological assays was confirmed by comparing antibodies eluted from blood spots with the equivalent plasma values in a series of samples from North Eastern Tanzania and by using blood spot-derived antibodies to estimate malaria transmission intensity in this site and for two localities in Uganda.

**Results:**

Antibodies in spots on filter paper and glass fibre paper had similar stabilities but blood was more easily absorbed onto filter papers than glass fibre, spots were more regular and spot size was more closely correlated with blood volume for filter paper spots. Desiccated spots could be stored at or below 4°C for extended periods, but were stable for only very limited periods at ambient temperature. When desiccated, recoveries of antibodies that are predominantly of IgG1 or IgG3 subclasses were similar. Recoveries of antibodies from paired samples of serum and of blood spots from Tanzania which had been suitably stored showed similar recoveries of antibodies, but spots which had been stored for extended periods at ambient humidity and temperature showed severe loss of recoveries. Estimates of malaria transmission intensity obtained from serum and from blood spots were similar, and values obtained using blood spots agreed well with entomologically determined values.

**Conclusion:**

This study has demonstrated the suitability of filter paper blood spots paper for collection of serum antibodies, and provided clear guidelines for the treatment and storage of filter papers which emphasize the importance of desiccation and minimisation of time spent at ambient temperatures. A recommended protocol for collecting, storing and assaying blood spots is provided.

## Background

When carrying out serological surveys, particularly in remote locations, it is of great advantage to have a method of collecting and storing blood samples which does not require that facilities for centrifugation are accessible, and which is relatively robust to irregular degrees of refrigeration, at least for short periods. One approach that seems to offer these advantages is to collect samples as dried blood spots, and to recover antibodies from the dried spots once transferred back to the laboratory. Blood spots have the advantage that they are quick, simple and inexpensive to prepare and to store, require very small blood volumes which can be obtained by finger- or heel-prick, and are likely to be more socially acceptable in cultural contexts in which larger volumes of blood are difficult to collect. Blood spots have been used routinely since the 1960s [1] for neonatal screening, initially for phenylketonuria, but subsequently for many other biochemical assays, including the assay of specific enzymes, determination of metabolites by mass spectrometry and, on occasion, for measuring antibody levels. More recently blood spots have been used as a source of DNA for screening for genetic abnormalities in newborns, for example for cystic fibrosis and haemoglobinopathies [2,3]. Blood spots have been used for monitoring antibodies against several viral [4-8], bacterial [9,10] and other [11-16] pathogens, storage of monoclonal antibodies[17] and, increasingly, for screening for HIV infection [18-20], both as a source of antibodies [9,21,22] and for virus detection by PCR. Dried blood spots have been particularly useful for isolating parasite DNA in mapping the spread of drug resistance in malaria parasites [23,24]

Although the stability of low molecular weight analytes in blood spots has been extensively studied [25] and guidelines have been produced [26] for blood spot collection, transport and storage together with recommendations and structures for quality control and quality assurance, the recovery of antibodies from dried blood spots has been less thoroughly investigated. Although small-scale studies of antibody stability in blood spots have been reported [27], these studies were not designed to be of predictive use in assessing storage conditions. In preparation for use of blood spots to derive serological measures of malaria transmission intensity [28], a thorough analysis of the stability of anti-malarial antibodies in blood spots has been undertaken, based on the well-established techniques used for determining the stability of biological reference materials [29-32]. This paper presents the results of these studies together with a validated protocol for collection, storage and use of blood spots for antibody quantitation.

## Methods

### Samples

Blood spots for stability studies and protocol optimisation were artificially constituted from fresh erythrocytes and stored heparinized plasma from hyperimmune donors from Brefet, The Gambia [33]. To determine the efficiency of antibody recovery from blood spots collected in the field, paired blood spots and serum samples were collected by finger prick from individuals attending the dispensary at Msitu wa Tembo, Lower Moshi in Northern Tanzania [34]. To further validate the final protocol, blood spots were collected onto 3 MM paper from individuals presenting at health facilities in Kabale and Rukungiri district in south west Uganda [35], excluding those with an active malaria infection. All studies were reviewed and approved by local ethical committees and within the London School of Hygiene and Tropical Medicine.

### Blood spot preparation and storage

For spot size measurements, fresh heparinized blood was spotted onto sheets of Whatman no 1, Whatman 3 MM (Whatman, Maidstone, UK) or glass fibre (Printed Filtermat A, Perkin Elmer, Beaconsfield, UK) filter paper in volumes from 1 μl to 45 μl. For antibody stability and recovery studies, reconstituted blood was prepared by mixing individual African malaria-hyperimmune plasma samples or a hyperimmune plasma pool or a European (non-exposed) negative control pool (all containing heparin) 1:1 by volume with washed European blood group O erythrocytes and immediately spotting 10 μl of this mixture onto Whatman 3 MM or glass fibre filter paper. Spots were allowed to dry at ambient temperature and relative humidity (RH) overnight.

Laboratory-generated blood spots and blood spots from Uganda were stored in individual self-sealing plastic bags (25 cm × 25 cm approx), which were then combined into sets and stored within three further successive plastic bags, the innermost of which contained approximately 3 g of self-indicating silica desiccant gel type III (Sigma). The bags were inspected regularly to confirm that the desiccant remained blue (RH < 20%), and the desiccant replaced if necessary.

Fingerprick blood samples from lower Moshi were collected into EDTA-coated microtainers and as blood spots on Whatman 3 MM paper and transported daily to the laboratory at Kilimanjaro Christian Medical College. Filter papers were refrigerated (2–8°C) in individual plastic bags with silica gel. Plasma was separated from packed red blood cells after centrifugation and stored at -20°C. All samples were assayed within 8 weeks of collection.

### Correlation between blood volume and spot area

Increasing volumes (1 μl – 45 μl) of fresh heparinized blood were pipetted (five replicates per volume) onto Whatman 3 MM, Whatman No 1 or Glass Fibre filter paper and the paper allowed to dry overnight at ambient temperature and RH. Next day the paper was optically scanned at 150 dots per inch (approx 5.9 dots per mm) in a desk-top scanner (Lexmark Model X5150, Lexmark, Marlow, UK) as 256-level grey-scale images. The spot areas were measured by integration using the program ImageJ (Rasband, W.S., ImageJ, U. S. National Institutes of Health, Bethesda, Maryland, USA, , 1997–2005). Pixel counts were converted to mm^2 ^and plotted against the volume of blood applied.

### Elution of antibodies from blood spots

Plastic bags containing blood spots were allowed to return to ambient temperature before opening. Discs approximately 2.5 mm in diameter were cut from the filter papers using a leather punch (Rolson Quality Tools, Twyford, UK). We found that this was best done by placing the papers on a 0.6 mm thick cardboard backing. Individual filter paper discs were transferred to individual wells of a 96 well titreplate (Greiner PS) and antibodies eluted with 150 μl PBS/0.05% (v/v) Tween20/0.05% (w/v) NaN_3 _at ambient temperature (23°C) overnight for 18 h with gentle mixing (rotary shaker at 2 revs per sec), giving a concentration of eluted serum proteins equivalent to a 1:100 dilution of the original blood. (i.e. approximately 1:200 with respect to plasma or serum, assuming a haematocrit of 50%).

### Antibody determination by ELISA

Microtitre plates (Immulon 4HBX, Thermo) were coated with recombinant MSP-1_19_.GST (to which antibodies are predominantly of the IgG1 subclass [36]) or MSP-2.GST (to which antibodies are predominantly IgG3 [36]) and blocked with 1% (w/v) skimmed milk powder. Samples were assayed as described previously[36,37] except that coating antigens, test samples and secondary antibody conjugate were each added in a total volume of 50 μl per well. Ten microliters of the antibody-containing eluate of each spot were added to individual wells of the coated and blocked microtitre plate together with 40 μl blocking buffer to give a final concentration of 1:1,000 with respect to the corresponding plasma sample. Each plate included a five-fold dilution series (1:50 to 1:156,250 final dilutions) of a standard African hyperimmune plasma pool. Bound antibodies were detected with either rabbit anti-human-IgG -HRP (Dako, Ely, UK), or sheep-anti-human IgG1 or IgG3-HRPconjugates (The Binding Site, Birmigham, UK) secondary antibodies and developed with *o*-phenylenediamine-H_2_O_2_.

A titration curve was fitted to the ODs obtained for the standard plasma dilutions by least squares minimisation using a three variable sigmoid model and the solver add-in in Excel (Microsoft), assuming an arbitrary value of 1000 Units/ml of antibody against each antigen in the standard pool. OD values for the spot extracts were converted to units/ml using this fitted curve.

Recoveries for blood spots were estimated as follows (full details in Additional file 1): serum or plasma ODs were converted to concentrations as above, the concentrations were multiplied by a recovery factor and then converted back to 'corrected' ODs – the ODs which would have been obtained if the serum or plasma had been more dilute. The value of the recovery factor was then optimized by weighted least squares minimisation comparing the actual ODs for the blood spots and the corrected OD values for serum or plasma, using the solver add-in in Excel™.

### Immunoglobulin stability studies

The stability of the immunoglobulin captured on Whatman 3 MM and GF filters was assessed by accelerated stability studies. Replicate sets of spots were prepared and triplicate spots stored at temperatures from 23°C to 65°C for between 1 and 16 weeks, at the end of which time they were transferred to -20°C and all samples were analysed together at the end of the study by ELISA. OD values for antibodies to MSP1_19 _and MSP-2 were converted to concentrations from the standard curve and for each temperature of storage the first-order rate constants, *k*, were calculated by fitting to an exponential model (ln(fraction of original remaining) versus time) in the statistical package Stata (Statacorp, College Station, Texas). This set of rate constants was then used to fit an Arrhenius equation [29] to derive the dependence of ln(*k*) on 1/T, the reciprocal of the absolute temperature. Values of ln(*k*) were weighted by the logs of the reciprocals of the variances of the first-order fits. Best estimated values and confidence intervals for different temperatures were calculated from this fitting exercize. For the glass fibre spots, the values were also fitted directly by least squares minimization without log transformation (which gave less weight to the most degraded samples) with essentially similar results.

### Calculation of malaria transmission intensity from antibody prevalences

Age-specific prevalence of anti-malarial antibodies is highly correlated with a number of measures of malaria transmission intensity [28]. To determine whether blood spots are a suitable source of antibodies for this purpose, blood spot eluates were tested (as above). To generate an OD cut-off value above which samples were deemed antibody positive, the distribution of OD values was fitted as the sum of two Gaussian distributions (a narrow distribution of seronegatives and a broader distribution of seropositives) using maximum likelihood methods. The mean OD of the Gaussian corresponding to the seronegative population plus three standard deviations was used as the cut-off for seropositivity (Cook, Drakeley, Griffin, Ghani and Corran; manuscript in preparation). The annual seroconversion rate, λ, and the annual rate of reversion to seronegativity, ρ, were estimated by fitting a simple model of the acquisition and loss of antibodies to the age-specific prevalence of the antibodies using maximum likelihood methods assuming a binomial distribution as in previous work [28]. The equivalent annual entomological inoculation rate (EIR) was then estimated using a calibration curve derived from previously determined values, (mostly from Tanzania but including two estimates from west Africa) [38].

## Results

### Relationship of spot size to blood volume

**Figure 1:**
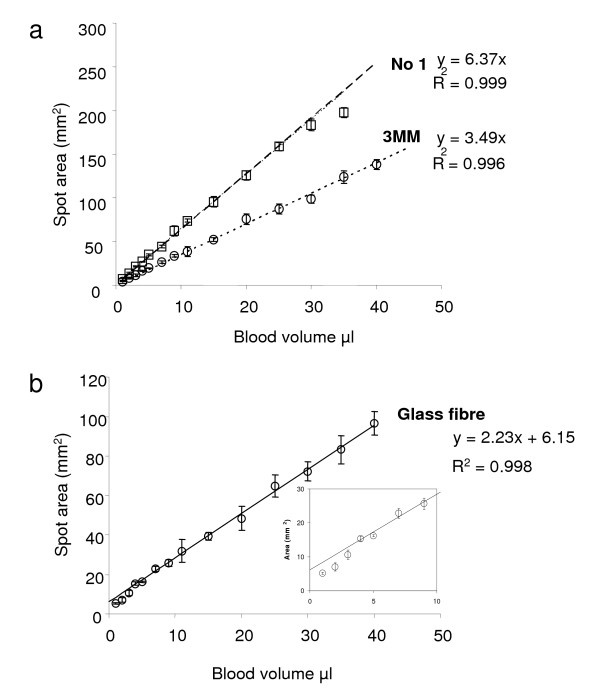
**Correlation between blood volume and blood spot area**. Increasing volumes (1 μl – 45 μl) of fresh heparinized blood were pipetted (five replicates per volume) onto Whatman 3 MM, Whatman No 1 or glass fibre filter paper, dried at ambient temperature and RH and optically scanned. Spot areas were measured by integration and plotted against the volume of blood applied. a) The relationship between volume and blood spot is linear over the entire range. A 2.5 mm diameter spot represents 0.77 μl and 1.4 μl of blood for No 1 paper and 3 MM paper respectively. Whatman No 1 filter paper (- -□- -) and Whatman 3 MM filter paper (.....○.......). b) Glass fibre paper. For blood volumes above 10 μl, spot size is linearly related to blood volume with a 2.5 mm diameter spot representing a volume of 2.2 μl of blood. For blood volumes below 10 μl (inset) the relationship between spot size and volume is unreliable.

For fresh blood, the relationship between total spot area and volume was linear for Whatman No 1 and Whatman 3 MM (Figure 1a) with gradients of 6.4 and 3.5 mm^2^/μl respectively. These correspond, for a 2.5 mm diameter spot, to volumes of 0.77 and 1.4 μl respectively. The ratio of these figures agrees well with the relative density of the two papers (Whatman No 1 : 9.0 mg/cm^2^; Whatman 3 MM :18.2 mg/cm^2^).

For blood spots on glass fibre paper the relationship between area and volume was less regular (Figure 1b). For volumes >10 μl, the relationship was linear with an increase in volume of 1 μl giving an increase in spot area of 2.23 mm^2^, however, for volumes below 10 μl the relationship was not co-linear with that for volumes above 10 μl.

### Blood spot quality

**Figure 2:**
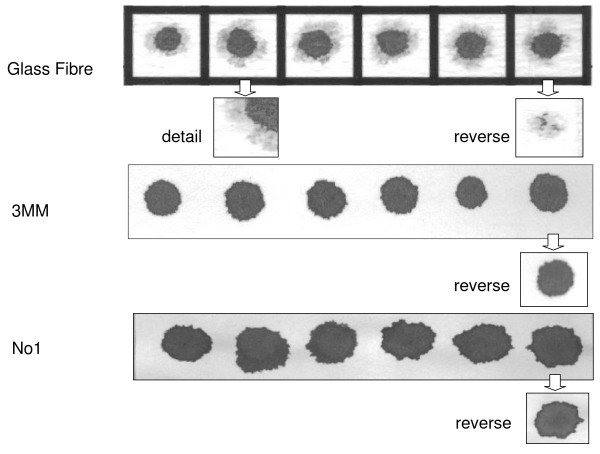
**Blood spot quality**. Fifteen μl of fresh blood were spotted onto glass fibre filter paper (top), Whatman 3 MM paper (middle) and Whatman No 1 (bottom) and scanned on both sides. For each paper, the first row of images represents the side of the paper to which blood was applied; the lower image on the extreme right hand is the obverse view of the spot immediately above. For the glass fibre paper, a four-fold magnification of one of the spots is also shown.

Both of the cellulose papers, 3 MM and No 1, wetted easily completely through the thickness of the paper, giving clean edges to the spot (Figure 2). The glass fibre paper was less readily wetted; blood drops did not consistently penetrate the full thickness of the paper and wetting at the edge of the spot was uneven (Figure 2). Spots on No 1 paper were less regular than on either of the other papers. Both 3 MM and No 1 were more robust to handle than glass fibre which tore easily and sometimes fragmented when discs were punched.

### Recovery of antibodies from blood spots

**Figure 3:**
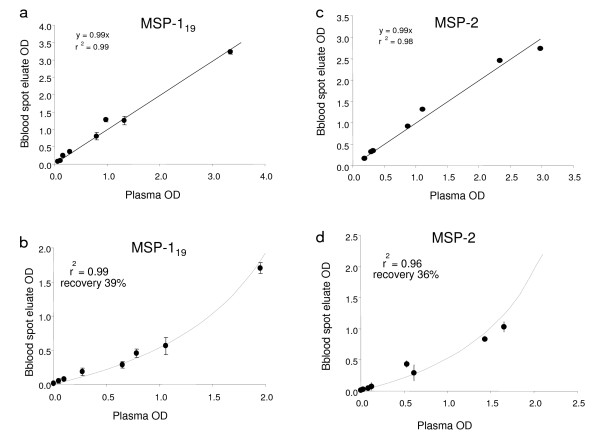
**Efficiency of recovery of antibodies from blood spots**. Comparison of OD obtained from paired plasma and blood spot eluates (each at an equivalent of 1:000 dilution) where blood spots were stored for 2 days (a,c) or 2 weeks at ambient temperature and RH (b,d). Samples were assayed for antibodies to MSP-1_19 _(a,b) and MSP-2 (c,d). Aliquots of 8 hyperimmune plasma samples were mixed with equal volumes of erythrocytes, spotted on 3 MM paper and dried overnight. The lines in a,c are the best fit least square straight lines, calculated recovery 99%. The dashed lines in c and d represent the least-squares best fit lines for the recoveries shown.

Eight hyperimmune African plasma samples were mixed with an equal volume of fresh erythrocytes and duplicate 10 μl aliquots were spotted onto 3 MM paper and dried. After 48 h at ambient temperature and RH, 2.5 mm discs were cut and incubated overnight at ambient temperature to elute the antibodies. The eluates were then assayed for total IgG to MSP-1_19 _and MSP-2 and, in one experiment, for MSP-1_19 _specific IgG1 and MSP-2-specific IgG3. The ODs obtained for the eluates were compared with those obtained for the corresponding plasma samples tested at the equivalent dilution. For both antigens the correlation between the OD values obtained for plasma and blood spot eluates was >0.98, with a recovery of >95% for total IgG (Figures 3a and 3c) and for IgG1 and IgG3. However, when blood spots were kept at ambient temperature and RH for two weeks, antibody recovery was much reduced, to approximately 40% of original concentration for total IgG for MSP-1_19 _and 35% for MSP-2 (Figures 3b and 3d). This variability in recovery prompted us to investigate the rate at which antibodies decayed under different storage conditions.

### Accelerated degradation of antibodies in blood spots

**Figure 4:**
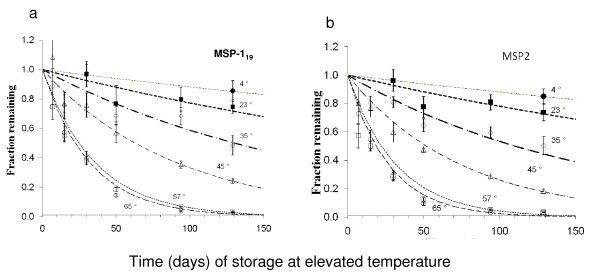
**Kinetics of loss of antibodies at different storage temperatures**. Blood spots were stored at + 23, 35, 47, 57 or 65°C for up to 130 days and then removed to -20°C until analysis. ODs were converted to antibody concentrations by reference to a standard curve. Lines represent the best-fit first order rate constants for each temperature.(a) MSP-1_19 _antibodies. (b) MSP-2 antibodies.

**Figure 5:**
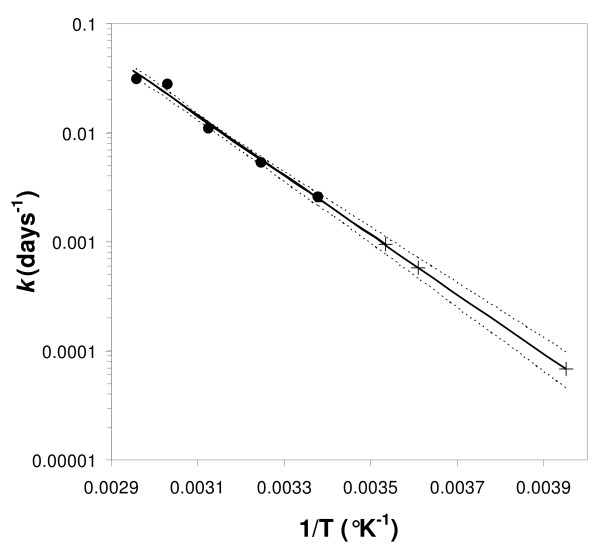
**Arrhenius plot of rates of loss of antibodies**. Rate of loss of antibody titre at each temperature (*k *days^-1^, the fitted first order rate constant) versus the reciprocal of the absolute temperature in °K (1/T). • measured points; + extrapolated values for 10°C, 4°C and -20°C;. The weighted best fit straight line is shown together with the 95% confidence limits.

Eluates were prepared from sets of blood spots from 3 MM and Glass Fibre paper that had been stored for differing lengths of time at temperatures between 23°C and 65°C and tested by ELISA for reactivity with MSP-1_19 _and MSP-2. Antibody concentrations were calculated by reference to the standard curve. The rate of decay of the antibody concentrations fitted a classical first-order kinetic plot of ln(c_t_/c_0_) (where c_t _is the concentration of antibody remaining at time t days at elevated temperature and c_0 _is the original concentration) (Figure 4). When used to construct an Arrhenius plot (Figure 5), the fitted values, weighted by the log of the reciprocal of the variance of the fit, could be used to predict rates of loss of antibody titre at other temperatures (Table 1). From this, we extrapolated the storage time and temperature at which 5, 10 or 20% (t_0.95_, t_0.9 _or t_0.8 _respectively) of the original antibody activity would be lost or, conversely, the length of time for which spots could be stored at varying temperatures in order to recover 80, 90 or 95% of the original antibody reactivity (Table 2). Antibodies to both MSP-1_19 _(which are predominantly of the IgG1 subclass[36]) and MSP-2 (predominantly IgG3[36]) decayed at the same rate (Figure 3 and Table 1).

**Table 1:** Measured and fitted values of first order rate constant *k *for (a) anti-MSP-1_19 _and (b) anti-MSP-2 recoveries from spots on 3 MM paper

(a) MSP1_19_					
Temp (°C)	Measured *k *(days^-1^)	Fitted *k *(days^-1^)	(95% CI)	t_1/2_	(95% CI) (years)
-20		0.00007	(0.00003–0.00014)	27.9	(13.1–59.4)
4		0.00058	(0.00037–0.00091)	3.3	(2.1–5.1)
10		0.00094	(0.00064–0.00138)	2.0	(1.4–2.9)
20		0.00202	(0.00153–0.00266)	0.94	(0.71–1.24)
23	0.0025789	0.00248	(0.00193–0.00319)	0.77	(0.59–0.99)
35	0.0053228	0.00564	(0.00477–0.00665)	0.34	(0.29–0.40)
47	0.0110235	0.01201	(0.01042–0.01385)	0.16	(0.14–0.18)
57	0.0281645	0.02258	(0.01882–0.02709)	0.08	(0.07–0.10)
65	0.0311997	0.03512	(0.02792–0.04417)	0.05	(0.04–0.07)

(b) MSP-2					
Temp (°C)	Measured *k *(days^-1^)	Fitted *k *(days^-1^)	(95% CI)	t_1/2_	(95% CI) (years)

-20		0.00007	(0.00003–0.00014)	28.8	(13.8–60.2)
4		0.00059	(0.00038–0.00091)	3.2	(2.1–5.0)
10		0.00096	(0.00067–0.00139)	2.0	(1.4–2.8)
20		0.00209	(0.00159–0.00273)	0.91	(0.70–1.19)
23	0.00248	0.00260	(0.00204–0.00332)	0.73	(0.57–0.93)
35	0.00629	0.00605	(0.00516–0.00709)	0.31	(0.27–0.37)
47	0.01342	0.01319	(0.01147–0.01517)	0.14	(0.13–0.17)
57	0.03154	0.02418	(0.02020–0.02895)	0.08	(0.07–0.09)
65	0.03723	0.03828	(0.03047–0.04809)	0.05	(0.04–0.06)

Measured and fitted values of *k *together with confidence limits are shown together with extrapolated values and confidence intervals for other selected temperatures and calculated half-lives

**Table 2:** Estimates of times (in years) for recoveries of antibodies in desiccated blood spots

	**Time (years) for original value to fall to :**		
**T (°C)**	**80%**	**90%**	**95%**
**-20°C**	9.0	4.2	2.1
**4°C**	1.1	0.5	0.2
**10°C**	0.65	0.31	0.15
**15°C**	0.44	0.21	0.10
**20°C**	0.30	0.14	0.07
**25°C**	0.21	0.10	0.05
**30°C**	0.15	0.07	0.03

Extrapolated times for total anti-MSP-1_19 _IgG to fall to a given percentage of initial concentration in desiccated blood spots on 3 MM paper at different temperatures.

In general, predicted stabilities for blood spots on glass fibre paper were slightly lower than for 3 MM paper, but for all temperatures the 95% confidence intervals for the two formats overlapped, so these differences cannot be considered statistically significant.

Overall, the study showed that antibodies in blood spots should be stable for extended periods of time (years) if kept desiccated at temperatures below 4°C (Table 2). When kept refrigerated (4–10°C) antibodies were highly stable for one to two months which would allow spots to be collected under field conditions and transferred in batches to long term storage at -20°C. However, antibodies degraded rapidly when stored at ambient temperature indicating that, ideally, blood spots need to be refrigerated within a week of collection.

**Figure 6:**
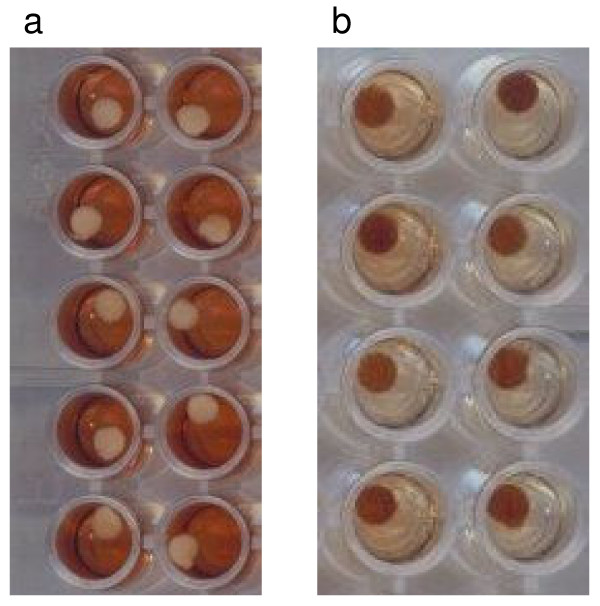
**Appearance of fresh and degraded blood spots**. Eluates from fresh blood spots (a) and spots stored for approximately 1 year at ambient temperature and humidity (b) are shown. In (a), complete elution of haemoglobin has resulted in a white filter paper disc in a red solution. In (b), little haemoglobin has been eluted so that the disc remains dark red and the solution pale.

The state of degradation of the blood spots could be roughly assessed by visual inspection of the eluates after overnight incubation. Since elution of haemoglobin from the filter papers correlates with elution of plasma proteins including immunoglobulin, filter paper discs from freshly made spots, or from spots stored at -20°C, are very pale (looking white against the haemoglobin-containing eluate when the plates are viewed from below) (Figure 6a), whereas discs made from heavily degraded spots remain reddish-brown against a pale background after reconstitution (Figure 6b). Thus, visual inspection can reveal something about the likely quality of the eluate and allows heavily degraded samples to be identified, and discarded, prior to ELISA assay.

### Comparison of serum and blood spots as sources of anti-malarial antibodies collected under field conditions

**Figure 7:**
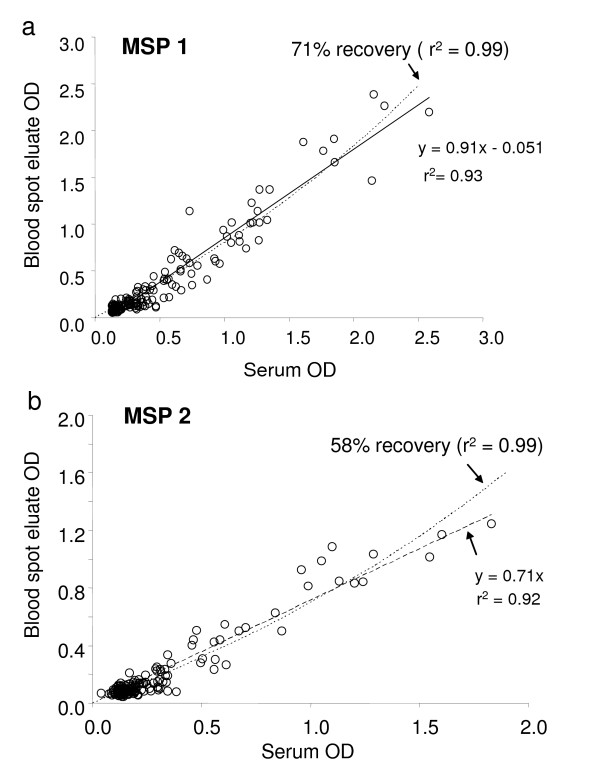
**Recovery of antibodies from blood spots and plasma in field studies**. Paired sets of serum and blood spots collected in Lower Moshi, Tanzania were collected and assayed for antibodies to MSP-1_19 _(a) and MSP-2 (b). The straight lines represent the least squares best fit; the curved dashed lines represent the least squares best fit lines where the recovery has been allowed to vary, giving a mean recovery of 71% for MSP-1_19 _and 58% for MSP-2.

Serum samples and eluates of contemporaneously collected blood spots, from 252 individuals living in Lower Moshi, Tanzania, were tested for anti-malarial antibodies by ELISA. The measured concentrations of antibodies in serum and in blood spot eluates were highly correlated for both MSP-1_19 _(r^2 ^= 0.93) and MSP-2 (r^2 ^= 0.92) (Figure 7). For MSP-1_19 _the straight line fit of ODs of antibody detected in blood spot eluates with the equivalent serum suggested 91% recovery (Figure 7a), although the best fit line allowing recovery to vary suggested a figure of approximately 70% (r^2 ^= 0.99). For MSP-2, however, the antibody recovery efficiency was noticeably lower (approx 70% based on OD or 60% based on titre; Figure 7b) suggesting that data obtained from blood spots can be compared qualitatively with data obtained from serum but that caution should be used when comparing these data quantitatively.

### Analysis of field samples

**Figure 8:**
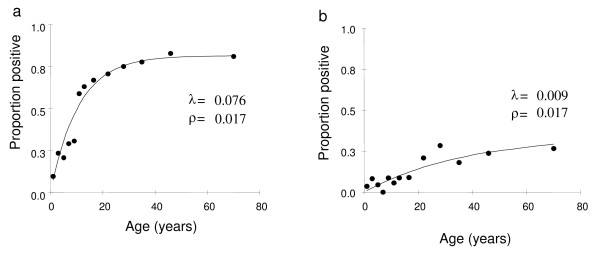
**Using blood spot eluates to estimate malaria transmission intensity in Uganda**. Observed (•) and estimated (line) age-specific prevalence of antibodies to MSP-1_19 _derived from blood spots obtained from parasite-negative individuals presenting at medical centres in (a) Rukungiri District (N= 1020 ; λ = 0.076; altitude 150–1,767 m) and (b) Kabale District (N= 958 ; λ = 0.009; altitude 1,948–2,420 m) Uganda.

Anti-malarial antibody concentrations determined from analysis of eluates of blood spots collected from 958 individuals of different ages in the Kabale district and 1020 individuals in the Rukungiri district of Uganda were assayed for anti-MSP-1_19 _antibodies. The samples were carefully desiccated and kept cool, and the appearance of the eluates from the spots was excellent (Figure 6). The proportion of seropositives for each age group was plotted (Figure 8). The estimated seroconversion rate (λ) for Rukungiri was 0.076 (SE 0.006), which corresponded to an EIR of 6.1 ib/p/y and agreed well with a recent entomological estimate of 6.0 ib/p/y [39]. For Kabale, the estimated λ was 0.009 (SE 0.002), corresponding to an EIR of 0.07 ib/p/y which is in reasonable agreement with a previous entomological estimate of 0.4 [40]. The common value of ρ was 0.017 (SE 0.003).

## Discussion

Blood spots collected onto filter paper are an attractive alternative to collection of plasma or serum for serological studies, particularly in remote field settings with limited laboratory facilities. Blood spots are an even more attractive option in the context of malaria studies where they are frequently already collected either for malaria diagnosis (as rapid diagnostic tests) or as a source of DNA for PCR-based detection and genotyping of parasites. However, whereas the ability to discern simply the presence or absence of parasites is frequently sufficient for DNA-based assays, antibody measurements tend to be of more value if information about the quantity of the antibodies present can be reliably obtained. This requires implementation of carefully validated protocols, for collection and storage of spots and elution of antibodies, designed to maximize the recovery of intact immunoglobulins.

The utility of the procedures presented here rests on the assumptions that the blood spot is evenly impregnated with blood, so that a disc of a standard diameter represents a defined volume of blood, and the adsorbed proteins can subsequently be eluted with high efficiency. A number of alternative substrates have been used for collecting blood spots and we have not been able to evaluate all of these here. Some of these substrates have been optimized for the recovery of DNA, but it is not clear that treatments to reduce nuclease activity and improve nucleic acid recovery are as beneficial for protein recovery. Of those that have been evaluated here, glass fibre mats and Whatman No 3 MM had high adsorption capacities while 3 MM and Whatman no 1 were robust for handling in a field environment. Glass fibre paper, however, was fragile, especially when punching discs, had inferior wetting characteristics and the efficiency of antibody elution was non-linear. Although antibody recoveries from No 1 paper were not systematically compared with those from 3 MM, limited experience suggests that recoveries are similar when the different adsorption capacities are allowed for. On balance, Whatmann 3 MM paper appears to offer the ideal combination of strength, wettability and adsorption. Moreover, comparison of amounts of antibodies recovered from freshly-prepared blood spots on 3 MM showed good correspondence with levels determined in the equivalent plasma.

The utility of this protocol also depends on establishing storage conditions under which immunoglobulins are stable in dried form, and can be quantitatively recovered from the paper at a later date. To estimate the stability of intrinsically stable preparations it is necessary to carry out accelerated stability testing at elevated temperatures, ideally under conditions where a substantial fraction of the activity has decayed during the period of measurement [30-32]. Stability testing is an integral part of pharmaceutical development [41,42], although most regimes are intended to address a relatively modest shelf-life. The choice of a first-order kinetic model is an empiric one, but a choice that appears compatible with experience of accelerated stability testing of lyophilized biological reference materials [29]. In the current case, it appears justified as a satisfactory approximation of the kinetic behaviour observed, though using higher temperatures than previously reported studies [25] in order to obtain adequate losses of activity. Encouragingly, this analysis demonstrates that, when the results are extrapolated below 23°C, it should be possible to obtain good quality antibody from blood spots kept for several years at -20°C and, more importantly, from blood spots stored for a few days at ambient temperature, and a few weeks at 4–10°C before either assay or transfer to -20°C.

The other major factor affecting the stability of antibodies in blood spots is the humidity at which they are stored. This was not examined in detail, partly because of the added experimental complexity of manipulating RH, but, more practically, because of the impossibility of monitoring and integrating changing levels of RH in field use. However, storage of spots for as little as two weeks at ambient humidity reduced the recovery of intact immunoglobulins by more than 50%, a far greater decrease than would have been expected if the spots had been scrupulously desiccated. Given the importance of keeping blood spots dry, the simple visual check provided by self-indicating silica gel appears to be the simplest if not the only practical method of monitoring storage humidity. Silica gel containing CoCl_2 _as indicator remains classified as safe for use in desiccation despite the recent classification of CoCl_2 _as being "possibly carcinogenic to humans" by the International Agency for Research on Cancer [43] and its classification as a carcinogen by the European Community. However, proprietary alternatives have recently been introduced (eg Sigma, Poole, UK #136767 ; Geejay, Sandy, UK ; Silgel, Telford, UK) which appear to change colour at RH <20%, and may therefore be equally suitable.

Although the rates of loss of antibodies to MSP-1_19 _and MSP-2 in the accelerated degradation study were similar, recoveries from field samples suggested that antibodies to MSP-1_19 _(predominantly IgG1) appeared to be more stable than antibodies to MSP-2 (predominantly IgG3). IgG3 antibodies have a greatly extended and unstructured hinge region [44,45] and it is possible that IgG3 antibodies are more susceptible to degradation by endogenous proteases when RH is not rigidly controlled. Given these observations, prudence suggests that the results of antibodies recovered from blood spots should not be combined with results from serum or plasma samples.

One unexpected advantage of this method of sample collection is that the quality of blood spots of questionable provenance (in terms of their storage conditions) can be judged visually by examining the colour of the filter papers discs after overnight incubation in elution buffer. Discs that have been properly stored, and from which there is a very high recovery rate of antibodies, turn from deep red to pale pink or white after incubation, indicating that the adsorbed proteins (including haemoglobin) have been eluted. By comparison, discs stored at high temperatures remain dark red, indicating that the indicator protein haemoglobin can no longer be readily eluted from the filter paper. When blood spots from different malaria field studies, which had been collected for diagnosis or DNA recovery and had been stored for extended periods at ambient temperature and humidity were examined, the eluates of spots which gave poor antibody recovery were uniformly pale in colour. Thus, whilst visual inspection may not be a fool-proof means of assessing blood spot quality it will allow seriously degraded samples to be identified and discarded prior to ELISA analysis.

Finally, the utility of blood spot collection of serum antibodies has been demonstrated by showing that it is possible to derive serological estimates of malaria endemicity from blood spot samples and that these serological estimates agree well with entomological data. Close agreement was observed between the serological and entomological data for Lower Moshi and for Rukungiri; agreement was less good for Kabale which may be due to the longer gap between the two estimates (the only estimate of EIR available was from 1996/7) or the inherent inexactitude of entomological assessments at low transmission [46,47].

## Conclusion

A protocol for collection of blood spots for serological use has been developed and validated. It is recommended that blood is spotted onto 3 MM paper and allowed to dry overnight at ambient temperature and humidity. The papers should then be interleaved with non-adsorbent paper (such as that used for interleaving photographic prints) or cardboard covers and immediately placed in a self-sealing plastic bag which is contained within a second bag containing desiccant. These bags should then be placed within two further self-sealing bags. Spots can be kept at ambient temperature for up to seven days without serious loss of activity provided they are desiccated. They can be kept, desiccated, for up to four weeks if refrigerated at 4°C or below. For storage of more than four weeks, the desiccated spots should be kept at -20°C. The desiccant should be regularly inspected and replaced if it changes colour. (A recommended protocol for collecting, storing and assaying blood spots is provided in Additional file 2).

## Competing interests

The authors declare that they have no competing interests.

## Authors' contributions

PC Conceived study, developed protocol, carried out and analyzed experimental work, drafted paper. JC developed protocol, participated in experimental work, drafted paper. TB conceived and carried out surveys in Tanzania. HL carried out surveys and assays in Tanzania. AM carried out surveys and assays in Tanzania. CL conceived, organized and carried out surveys and analyses in Uganda. JC supervised and participated in planning and execution of work in Uganda. TA assisted in supervision, planning and execution in Uganda. JG devized fitting algorithms, provided statistical advice. AG devised fitting algorithms, provided statistical advice. CD supervized and participated in surveys and assays in Tanzania and analysis of data, drafting paper. ER supervized work and drafted paper. All authors read and approved the final manuscript.

## Supplementary Material

Additional File 1 **Estimation of recoveries from ODs**. Outline of methods used to calculate relative recoveries from two sets of ODs of same samples. Click here for file

Additional File 2 **Protocol for obtaining, storing and reconstituting blood spots for serological purposes**. A full recommended protocol for Preparing, sampling and storing blood spots for serological use, and instructions for reconstituting and assaying antibodies by ELISA. Click here for file
