# Supplementary material for: Dried blood spots as a source of anti-malarial antibodies for epidemiological studies
Source: Malar J. 2008 Sep 30;7:195. doi: 10.1186/1475-2875-7-195 (PMC2567984; doi:10.1186/1475-2875-7-195)
Supplement: Additional File 1 — Estimation of recoveries from ODs. Outline of methods used to calculate relative recoveries from two sets of ODs of same samples. [file 1475-2875-7-195-S1.pdf]

Supplementary file I

Estimation of recoveries

Calculation of concentration from OD

The standard dilution curve was fitted to the ligand-binding equation :

$$A = A_{max} \bullet c/(c+k) \qquad \text{eqn 1}$$

Where A = OD measured (blank-corrected), A_{max} is the OD when the coated antigen is completely saturated with antibody (ie at infinite antibody concentration), c is the concentration of the antibody in the diluted serum or plasma =C/D where C is the undiluted concentration and D is the reciprocal of the dilution (ie 1000 if the dilution is 1:1000). *k* is the value of c when A = $A_{max}/2$. (Fig 1)

Figure 1

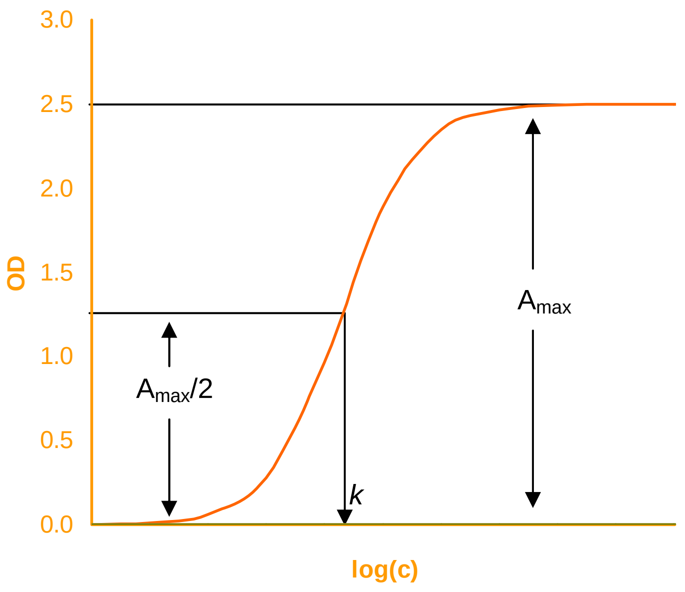

The concentration, s, of any sample, in terms of the standard, can now be read off this curve or estimated using the rearranged relationship :

$$s = k/(A_{max}/A - 1) \qquad \text{eqn 2}$$

If the standard is assigned an arbitrary value of 1000 antibody units, then *k* and *s* will be in these units, and *s* is the concentration of the diluted sample.

Calculation of ODs from corrected concentrations

If it is now assumed that the recovery of the eluate from blood spots is reduced by a factor *r*, so that the actual concentration of the final spot eluate dilutions analysed is not *s* but $r \bullet s$, then the expected ODs can be estimated by :

$$A' = Amax \bullet r \bullet s/(r \bullet s + k) \text{ (using eqn 1)}$$

Substituting in eqn 1and rearranging :

$$A' = A_{max} \bullet r/(A_{max}/A + r - 1) \qquad \text{eqn 3}$$

Where A’ is the estimated OD value with a recovery of *r* when the original OD is A. Where A is very small this simplifies to $A' = A \bullet r$. When $A \approx A_{max}$ this simplifies to $A' = A_{max}$. (Fig 2)

Figure 2

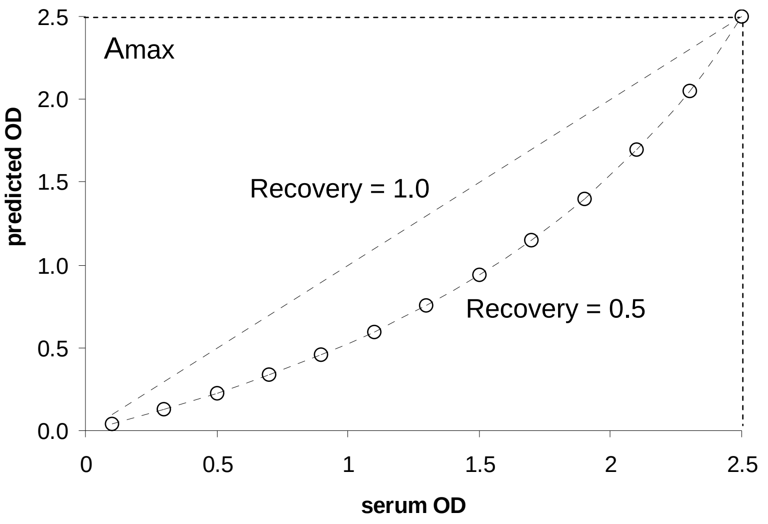

To estimate *r*, the ODs of sera modified by this equation are least-squares fitted to the actual values obtained from blood spots, varying *r* and weighting the squares of the differences of predicted and actual values by the reciprocal of the serum OD (which partially compensates for the fact that residuals will be maximum for ODs around 1-1.5, and least squares fitting will tend to overemphasise these values at the expense of low values)
