# Supplementary material for: Dried blood spots as a source of anti-malarial antibodies for epidemiological studies
Source: Malar J. 2008 Sep 30;7:195. doi: 10.1186/1475-2875-7-195 (PMC2567984; doi:10.1186/1475-2875-7-195)
Supplement: Additional File 2 — Protocol for obtaining, storing and reconstituting blood spots for serological purposes. A full recommended protocol for Preparing, sampling and storing blood spots for serological use, and instructions for reconstituting and assaying antibodies by ELISA. [file 1475-2875-7-195-S2.pdf]

Additional File II

Protocol for obtaining, storing and reconstituting blood spots for serological purposes

A full recommended protocol for Preparing, sampling and storing blood spots for serological use, and instructions for reconstituting and assaying antibodies by ELISA.

A. Preparation of filter papers

Materials :

- Stapler with staples, pen for marking, scissors
- Whatman 3MM filter paper (available as 20 x 20 cm squares or as 2 cm x 100 m rolls from GE Healthcare, Little Chalfont, Bucks, UK)
- Thin card : that used for document folders or for packaging Immulon 4 plates (see below) is suitable
- Labels (adhesive) – optional

1. Cut strips of Whatman 3MM paper 7 cm x 2 cm approx.
2. Cut a protective cardboard cover approximately 16 x 2.5 cm
3. place a strip of filter paper and a strip of cardboard end-to-end and overlapping by 0.5 cm and staple together
4. fold down the stapled part so that the filter strip lies down the cardboard strip
5. fold up the bottom of the cardboard strip over the filter paper to form a protective cover

Details of the donor can be written on the back of the cover, and/or a label or bar code stuck on.

B. Collection of Blood Spots

Materials :

- 3MM filter paper strips prepared as above
- Sterile Blood lancets
- Swabs
- Methylated Spirits (95% v/v ethanol)
- Clean Disposable gloves
- Sharps bin

Procedure :

1. Explain to patient/parent what you are about to do
2. Label the filter paper
3. Wipe the heel or thumb of child or finger of preference of adult with a swab dampened in methylated-spirits and dry with dry swab
4. Prick the site with a blood lancet and wipe off the first drop of blood
5. Collect two drops of blood as separate spots on the filter paper (approximately 20 µl) : check that the blood spots wet the full thickness of the paper
6. Discard the lancet into the sharps bin
7. Allow the spots to dry at ambient temperature overnight.

C. Storage of blood spots

Materials :

- Silica Gel desiccant : eg Sigma, Poole, Dorset, UK No. #136767, Geejay Chemicals, Sandy, UK ; Silgel, Telford, UK. There is a useful discussion of silica gels at http://www.geejaychemicals.co.uk/usingsachets.htm, and the latter sell 3 g bags of self-indicating Si gel
- Plastic self-sealing bags, at least 10cm x 10cm : self sealing freezer bags for food storage are suitable
- Rubber bands
- Plastic airtight storage boxes 5-10*l*
- Relative Humidity (RH) indicator strips (optional) (6 spots 10%-60% Geejay Chemicals, Sandy, UK)

Procedure :

1. When thoroughly dry, combine 10-20 spot folders together and secure with rubber band
2. Place the spots in a plastic bag. Roll up (to expel air) and seal
3. place rolled up bag within another : several sets of spots may be included in the one bag at this stage
4. Add 1 teaspoon (~3 g) (or a 3 g bag) of self-indicating silica gel – increase this amount if including more than 20 spots
5. Roll up and seal second bag
6. Place within third bag, roll up and seal.
7. Combine bags in an airtight container – a food storage box is suitable
8. Add a RH indicating strip (optional)
9. Keep as cool as possible; transfer to +4°C storage within 1 week
10. Inspect weekly and replace desiccant as necessary
11. Transfer to -20°C within 1 month
12. Inspect regularly : when desiccant indicates RH>20%, warm back to ambient, replace desiccant and return to -20°C
13. Always allow spots to warm back to ambient before opening.

D. Reconstitution of blood spots

Materials :

- Low binding 96-well flat-bottomed plates (e.g. Greiner bio-one cat no. 655-101)
- Acetate plate sealing film (e.g. Thermo Acetate Plate Sealers Part No. 3501)
- Thin card (e.g. thin document folder, card used for packing Immulon 4 plates)
- Leather punch 2.5 mm diameter (Rolson Quality Tools, Twyford, UK or Homebase, UK)
- Tweezers
- Cocktail sticks for removing punched circles from cutting piece.
- 12 or 8 channel 50-250µl pipette with tips and solvent troughs
- PBS-T-azide : 20 mM sodium phosphate-0.15M NaCl-0.05% (v/v) tween 20-0.1% (w/v) NaN_3 pH 7.2 (WARNING : Sodium azide is a poison, and azides are reactive with metals, forming explosive metal azides : ascertain and comply with local safety requirements concerning handling and disposal)
- Clean surface, e.g. Benchkote™

Procedure

1. Remove spots from cold storage and allow to warm to ambient temperature
2. Prepare a template of sample positions to be filled in as you proceed
3. Label a plate
4. Open the spots and separate
5. From the first paper cut a 2.5 mm diameter circle with the punch against the cardboard as backing (this ensures the punch will stay sharper for longer)
6. With a suitable probe (ie a cocktail stick), dispense the excised circle onto the working surface, separate from any cardboard which has also been cut out, and transfer carefully to the first well of the plate with the tweezers
7. Update the template with the sample details
8. Proceed until all wells on the plate have blood spot circles.
9. Set the pipette to 150 µl and dispense 150 µl PBS-T-NaN_3 into each well (it doesn't matter that the spots float on the surface)
10. Seal the plate with a sealing film
11. Shake gently on a rotary shaker at 1-2 rev/sec overnight at ambient temperature
12. Next day inspect the plate: the circles of filter paper should appear white and the solution pink/red in all cases. Reject samples where the circle is still red and the solution pale.
13. Plates may now be stored, sealed, at 4°C for up to 2 weeks until required: inspect every few days in case the seal starts to come away from the plate.

E. ELISA of antibodies against specific malaria antigens

Materials

- 96 well ELISA plates : Immulon 4HBX (Thermo)[the thin card included with each pack of 10 is very suitable for cutting out circles against – see above]
- 8 channel pipettes 50-250 µl and 5-50 µl
- Single channel pipettes 2-20 µl, 50-250 µl, 200-1000 µl
- Plastic boxes, 5*l*, for washing plates
- Troughs
- Skimmed milk powder
- **A**nti-Human-IgG-conjugate (HRP or AP), previously titrated
- Substrate and stop solution (will depend on conjugate)

Reagents

- Coating buffer : 0.2 M $NaHCO_3/Na_2CO_3$ pH 9.3
- PBS-T : 20 mM sodium phosphate-0.15M NaCl pH 7.2 (make up as 10x concentrate) with 0.05% (v/v) Tween-20 (ad after dilution)
- Blocking buffer : PBS-T with 1% (w/v) skimmed milk powder
- Substrate buffer : dependent on conjugate
- Stop solution : dependent on substrate

Procedure :

1. Coat ELISA plates with malaria antigens, normally at a concentration of 0.5 or 1.0 µg ml in coating buffer, 50 µl/well
2. Incubate overnight covered and sealed in a plastic bag at 4°C or wrapped in cling film.
3. Wash x3 PBS-T
4. Add 200 µl blocking buffer to each well and block ambient temperature 3h
5. Wash x3 PBS-T
6. Pat dry. Set up templates so that each plate contains a blank, a positive and a negative serum in duplicate, and each batch of plates of the same antigen contains a 3- or 5-fold titration curve of a standard positive serum to give a calibration curve For the Spot samples, it is usually best to arrange for 5

columns to be analysed in duplicate on each plate – ie columns 1-5 supply columns 1-10 of plate A (column 1 to columns 1&2, column 2 to 3&4 etc), columns 6-10 supply columns 1-10 of plate B. Columns 11&12 are reserved for controls and standards.

7. Add standards and controls to plates, 50 µl/well diluted in blocking buffer
8. *Either* : pipette 40 µl blocking buffer into each Spot well, then transfer 10 µl of each well, a column at a time, in duplicate, replicating for extra antigens *Or* : transfer the 10 µl of eluate first and add the 40 µl of blocking buffer afterwards.
9. Incubate overnight 4°C
10. Next day wash x5 PBS-T
11. Add 50 µl appropriately diluted anti-Human-IgG-conjugate in blocking buffer to each well
12. Incubate ambient temperature 3h
13. Wash x5 PBS-T, drain well
14. Make up substrate according to manufacturers instructions
15. Add 100 µl substrate to each well
16. Cover plates and place in dark to develop
17. After 15 min check progress, and stop reaction with stop solution when sufficiently developed
18. Read plate in plate reader at appropriate wavelength

F. Data Handling (outline – most is handled with a combination of templates and Excel macros)

1. Load each plate into a separate Excel Worksheet
2. Examine the duplicates on each plate : reject ODs >0.2 which differ by more than a factor of 1.5
3. Subtract the appropriate blank value from each OD and average
4. Scale each OD to the plate positive mean average = 100
5. Fit the standard dilution curve to a sigmoid 3-parameter model to derive titres

Data may now be carried forward and added to the main database for estimating positive cutoffs and fitting age-specific seroprevalence curves.
